# The Role of Intravitreal Anti-VEGF Agents in Rabbit Eye Model of Open-Globe Injury

**DOI:** 10.1155/2021/5565178

**Published:** 2021-04-15

**Authors:** Xiao Zhao, Han Han, Yinting Song, Mei Du, Mengyu Liao, Xue Dong, Xiaohong Wang, Ferenc Kuhn, Annette Hoskin, Heping Xu, Hua Yan

**Affiliations:** ^1^Department of Ophthalmology, Tianjin Medical University General Hospital, Tianjin, China; ^2^Laboratory of Molecular Ophthalmology, Department of Pharmacology and Tianjin Key Laboratory of Inflammation Biology, School of Basic Medical Sciences, Tianjin Medical University, Tianjin, China; ^3^Helen Keller Foundation for Research and Education, Birmingham, AL, USA; ^4^Save Sight Institute, The University of Sydney, Sydney, NSW, Australia; ^5^Lions Eye Institute, University of Western Australia, Perth, Australia; ^6^Centre for Experimental Medicine, School of Medicine, Dentistry & Biomedical Sciences, Queen's University Belfast, Belfast, UK

## Abstract

**Purpose:**

To evaluate the effects of intravitreal anti-VEGF agents in a rabbit model of open-globe injury (OGI).

**Methods:**

OGI was induced in the right eyes of 75 Belgian rabbits by making 5 mm circumferential incision placed 6 mm behind the limbus. The rabbits were divided into 4 groups: control (*n* = 5), OGI group (*n* = 40), and intravitreal Ranibizumab and Conbercept (*n* = 15 each). Ranibizumab or Conbercept was injected into the vitreous at 0.5 hours, 3 days, or 7 days. Vitreous fluid was collected, and levels of growth factors and cytokines were measured by enzyme-linked immunosorbent assay (ELISA). On day 28 after OGI, B scan examination and histological examination were performed to evaluate intravitreal proliferation and formation of epiretinal fibrosis.

**Results:**

Vitreous levels of vascular endothelial growth factor (VEGF), platelet-derived growth factor (PDGF), transforming growth factor-beta (TGF-*β*), and plasminogen activator inhibitor-1 (PAI-1) were significantly increased in rabbit eyes after OGI. Compared to eyes in OGI group, anti-VEGF treatments significantly reduced these growth factors and cytokines. Among the 7 eyes examined from each group for intravitreal proliferative changes, they were found in 7 of 7 (100%) in OGI group and were decreased by Ranibizumab and Conbercept to 5 of 7 (71.4%) and 4 of 7 (57.1%), respectively. Both Ranibizumab and Conbercept inhibited epiretinal scar formation at the wound site, with Conbercept showing the greatest effect (maximal length of scar (*L*), *L*_OGI_ = 503 ± 82.44 *μ*m, *L*_Ranibizumab_ = 355 ± 43.66 *μ*m, and *L*_Conbercept_ = 250.33 ± 36.02 *μ*m).

**Conclusion:**

Anti-VEGF treatments after OGI significantly attenuated the upregulation of growth factors and cytokines in the vitreous and prevented intravitreal proliferation and epiretinal scar formation and thus may protect against the development of posttraumatic complications such as proliferative vitreoretinopathy (PVR).

## 1. Introduction

Ocular trauma is the major cause of vision loss in children and young adults resulting in unilateral blindness of almost 19 million people worldwide [1]. Mechanical ocular trauma can be divided into open-globe injury (OGI) and closed-globe injury (CGI), depending on whether the integrity of the globe is violated [2]. An OGI or severe CGI often leads to serious complications including proliferative vitreoretinopathy (PVR), particularly when accompanied by retinal detachment (RD) or vitreous hemorrhage (VH) [3]. PVR occurs in 5–10% of all rhegmatogenous retinal detachments (RRD), but the incidence is estimated to increase in OGIs to approximately 50% [4]. Eyes with PVR are at high risk of developing late tractional RD and are associated with poor visual prognosis and ocular morbidity [5]. Despite advances in vitreoretinal surgery, PVR remains challenging to manage. Current pharmacologic strategies to prevent PVR formation are primarily anti-inflammation and antiproliferation treatments [6], and despite some promising results in animal models of PVR, there are currently no proven effective medical therapies for the treatment or prevention of PVR [7].

PVR is an exaggerated vitreoretinal wound-healing response that mainly consists of three overlapping phases: inflammation, cell proliferation, and extracellular matrix (ECM) formation and remodeling [8]. Previous studies have confirmed that a retinal break that exposes the retinal pigment epithelial (RPE) to the vitreous cavity is likely necessary for the development of PVR [9]. Increased production of growth factors and cytokines, presumably secreted by infiltrating immune cells, may make contact with intraretinal cells like RPE cells and initiate the cellular responses leading to PVR development [8].

Several growth factors, including platelet-derived growth factor (PDGF), vascular endothelial growth factor (VEGF), and transforming growth factor-beta (TGF-*β*), are elevated in PVR vitreous [10–12]. Among these, TGF-*β* has been shown to be a potent chemoattractant inducing RPE epithelial-to-mesenchymal transition (EMT), a process that transforms RPE into mesenchymal fibroblastic cells and induces ECM protein synthesis [11, 12]. PDGF plays a crucial role in PVR by promoting the fibrotic proliferation process [13]. In addition, coagulation factors like plasminogen activator inhibitor-1 (PAI-1) are also implicated in PVR by inhibiting fibrin degradation and promoting ECM accumulation [14, 15].

Anti-VEGF agents such as Ranibizumab and Conbercept are well-established therapies for the treatment and prevention of neovascular ocular diseases, like proliferative diabetic retinopathy (PDR) and choroidal neovascularization (CNV) [16, 17]. However, recent researches showed Ranibizumab had a potential antifibrotic effect in experimental PVR models [18, 19], while the underlying mechanisms are unknown. As previously mentioned, overexpressions of growth factors and cytokines after ocular trauma drive the cellular processes leading to PVR development and, thus, are thought to be pivotal in the pathogenesis of PVR. Therefore, antiproliferative agents that target these growth factors and cytokines may help reduce the risk for the development of PVR. In the current study, we evaluated the potential antiproliferation effect of intravitreal anti-VEGF agents in OGI animal models.

## 2. Materials and Methods

### 2.1. Animals

Belgian rabbits were handled in accordance with the Association for Research in Vision and Ophthalmology (ARVO) Statement for the Use of Animals in Ophthalmic and Vision Research and kept in the Experimental Animal Laboratory of Tianjin Orthopedic Institute. The experiments were approved by the Care and Use of Laboratory Animal Committee of Tianjin Medical University. The animals were acclimatized for 1 week prior to the experiment.

### 2.2. Open-Globe Eye Injury Model

Healthy Belgian rabbits (3–6 months old, 2–2.5 kg) were divided into 4 groups: control group, OGI group, OGI with intravitreal Ranibizumab group, and OGI with intravitreal Conbercept group. Before OGI, general anesthesia was given via intraperitoneal injection of 3.5 mL/kg chloral hydrate (10%), followed by topical anesthesia with oxybuprocaine hydrochloride drops (Benoxil, 4 g/L, Santen, Japan). The OGI model was established by making a 5 mm penetrating wound circumferentially 6.0 mm behind the limbus at the supratemporal quadrant of the right eye, and the wound was sutured 0.5 hours later with aseptic technique. For anti-VEGF treatments, a single dose of 0.25 mg Ranibizumab (25 uL, Genentech, Inc., and Novartis International AG, Basel, Switzerland) or Conbercept (25 uL, Chengdu Kanghong Biotech Co., Ltd., Sichuan, China) was injected into the vitreous of the eyes after OGI at indicated time point (0.5 hours, 3 days, or 7 days), for each time point, *n* = 5 animals. Levofloxacin eye drops (0.5%, Santen, Japan) were used three times a day for three days before and after the eye injury and intravitreal injections. Two eyes in OGI group were excluded for endophthalmitis as we observed hypopyon or intravitreal empyema about 2 weeks after OGI. No injury was induced in the left eye.

### 2.3. Ophthalmic Examinations and B Scan

Regular ophthalmic examinations were performed before and 3, 7, 14, 21, and 28 days after OGI. A slit lamp biomicroscope was used to observe the anterior segment, as well as any signs of inflammatory responses or uveitis. Indirect ophthalmoscopy was conducted to observe the changes of vitreous and retina, such as intraocular inflammation, vitreous hemorrhage, and retinal detachment. Intraocular pressure (IOP) was checked in all eyes. B scan ultrasonography (MEDA Co., Ltd., Tianjin, China) was performed in all four groups at 28 days after injury (*n* = 7 rabbits in each group). The intravitreal proliferative responses were assessed in accordance with the grade scale listed in Table 1 [20, 21]. The eyes scored at stage 1 or worse were considered to have a presence of intravitreal proliferation.

### 2.4. Collection of Vitreous Samples and Analysis

For OGI group, samples of vitreous fluid (∼100 *μ*l) were collected at 0.5, 1, 3, 7, 10, 14, 21, and 28 days after OGI by 24G syringes (*n* = 5 rabbits for each time point, for a total of 40 rabbits). For OGI with anti-VEGF treatment groups, vitreous fluid was collected at 7 days after a single intravitreal injection of anti-VEGF agents at indicated time points as described above. Vitreous samples collected from age-matched Belgian rabbits (*n* ≥ 5) without OGI induction were used as control. Vitreous samples were centrifuged for 15 minutes at 1000×g at 4°C, and supernatant was then stored in −80°C for further testing. Samples contaminated with blood were excluded. The levels of VEGF, PDGF, TGF-*β*, and PAI-1 in vitreous were measured using ELISA kits (Shanghai Enzyme-linked Biotech Co., Ltd., Shanghai, China) according to the manufacturer's instructions.

### 2.5. Histopathological Examinations

#### 2.5.1. Gross Exam and Masson Trichrome Staining

The animals were sacrificed at 28 days after OGI with an overdose intravenous administration of 2% pentobarbital, and the eyes were enucleated for histological analysis. Enucleated globes were fixed in 4% paraformaldehyde (PFA, pH 7.4) for 2 hours; then the anterior segments were removed, and the remaining eye cups were further fixed in 4% PFA for 36–48 hours and then were embedded in paraffin. The maximal length of epiretinal scar at the wound site was measured with vernier caliper. Gross view was photographed under stereoscope, and the scar area was analyzed using ImageJ2 analysis software. An 8 *μ*m thickness cross section through the optic nerve was prepared. Masson's trichrome staining was performed to stain collagen and fibrosis in rabbit eyes with OGI using Masson's trichrome staining kit (Solarbio Life Science, Beijing, China), and the volume of collagen fraction in the wound site of the retina was analyzed by ImageJ2 analysis software (collagen volume fraction = the collagen area divided by total area).

#### 2.5.2. Immunohistochemistry Staining

Immunofluorescence staining of alpha smooth muscle actin (*α*-SMA), a marker for myofibroblasts [22], was performed to further confirm formation of fibrosis at the wound site in rabbit eyes with OGI. The paraffin sections were heated at 55°C for 30 minutes; then xylene dewaxing and gradient ethanol hydration were performed. Following antigen retrieval with citric acid solution and microwave heating, the sections were blocked and permeabilized in 5% normal goat serum +0.3% Triton X-100 for 1 hour and were incubated with primary anti-(*α*-SMA) antibody (Cat. no. A2547, Sigma-Aldrich, St. Louis, MO, USA) overnight at 4°C. After washing with phosphate buffer saline (PBS), the slides were incubated with fluorescein isothiocyanate- (FITC-) conjugated secondary antibody for 1 hour at room temperature. After counterstaining with 4′,6-diamidino-2-phenylindole (DAPI), the stained sections were visualized under a fluorescence microscope (Type 108, Nikon, Japan).

### 2.6. Statistical Analysis

All experiments were performed at least 3 times. The mean and standard deviation (SD) were calculated and analyzed using SPSS version 22.0 (SPSS, Inc., Chicago, IL). Data were analyzed statistically using two-tailed one-way analysis of variance (ANOVA) test. *P* < 0.05 was considered statistically significant.

## 3. Results

### 3.1. Upregulation of VEGF and Intraocular Cytokines in the Rabbit Model of Open-Globe Injury

**Figure 1:**
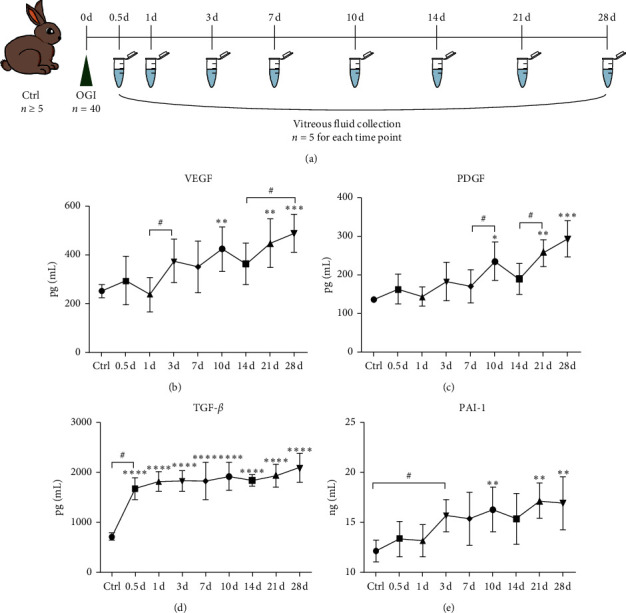
Changes of VEGF, PDGF, TGF-*β*, and PAI-1 levels in the vitreous fluid of rabbit eyes at different time points after open-globe eye injury. (a) Flow chart of OGI models and vitreous fluid collection. In control group, *n* ≥ 5; in OGI group, *n* = 40; and the vitreous fluid was collected at 0.5, 1, 3, 7, 10, 14, 21, and 28 days after OGI; for each time point, vitreous fluid was collected from 5 eyes in OGI group. ((b)–(e)) ELISA results showed that vitreous levels of VEGF, PDGF, TGF-*β*, and PAI-1 were significantly increased in rabbit eyes after OGI. Data are mean ± SD, *n* = 5. ^*∗*^*P* < 0.05, ^*∗∗*^*P* < 0.01, ^*∗∗∗*^*P* < 0.001, and ^*∗∗∗∗*^*P* < 0.001 versus control by one-way ANOVA with Tukey's multiple comparisons test; ^#^*P* < 0.05, comparisons among groups by ANOVA with LSD multiple comparisons test.

Compared to control group, concentrations of VEGF, PDGF, TGF-*β*, and PAI-1 in the vitreous fluid of the OGI group were all elevated (Table S1). VEGF increased significantly during 1–3 days and 14–28 days after injury (*P*_1-3*d*_ = 0.014, *P*_14-28*d*_ = 0.022) (Figure 1(b)), and PDGF increased significantly during 7–10 days and 14–21 days after injury (*P*_7-10*d*_ = 0.018, *P*_14-21*d*_ = 0.012) (Figure 1(c)). TGF-*β* levels peaked at 0.5 days after injury and maintained high levels throughout all the time points after injury (Figure 1(d)), and PAI-1 increased significantly during 0–3 days after injury (*P* = 0.005) (Figure 1(e)). The trend of all the cytokines showed significant increase over the 4 weeks' duration of the experiment.

### 3.2. Fundus Examinations and B Scan Ultrasonography of Rabbit Eyes after Open-Globe Injury

**Figure 2:**
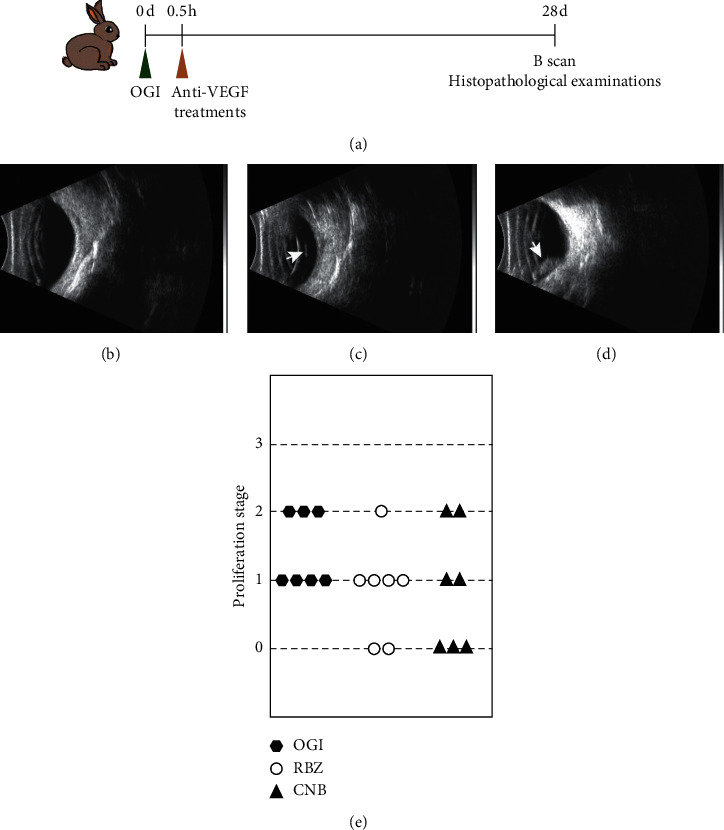
B-ultrasonic exam of rabbit eyes at 28 days after open-globe injury. (a) For anti-VEGF treatments, Ranibizumab or Conbercept was injected intravitreally at 0.5 hours after OGI. B scan and histopathological examinations were performed 28 days after OGI. (b) There was no abnormal echo in normal rabbit eyes. (c) Vitreous opacity and strands (white arrow) were detected in rabbit eyes with stage 1 intravitreal proliferation. (d) Vitreous opacity and epiretinal membrane formation (white arrow) were observed in rabbit eyes with stage 2 intravitreal proliferation, without any sign of retinal detachment. (e) Distribution diagram of proliferation stage in OGI, RBZ, and CNB groups. RBZ, OGI with intravitreal Ranibizumab; CNB, OGI with intravitreal Conbercept.

Fundus examinations were performed at 3, 7, 14, 21, and 28 days after OGI to observe the changes in the fundus. There was no retinal detachment in OGI and OGI with anti-VEGF treatment groups over 4-week period of the experiment. Furthermore, the intravitreal proliferative responses scored by B scan ultrasonography are shown in Figures 2(b)–2(e). In OGI group, 7 of 7 (100%) eyes presented intravitreal proliferation, while in OGI treated with Ranibizumab or Conbercept, the number of eyes with intravitreal proliferation decreased to 5 of 7 (71.4%) and 4 of 7 (57.1%), respectively, indicating an inhibitory effect of anti-VEGF agents on developing intravitreal proliferation after ocular trauma.

### 3.3. Gross Examination of Rabbit Eyes after Open-Globe Injury

**Figure 3:**
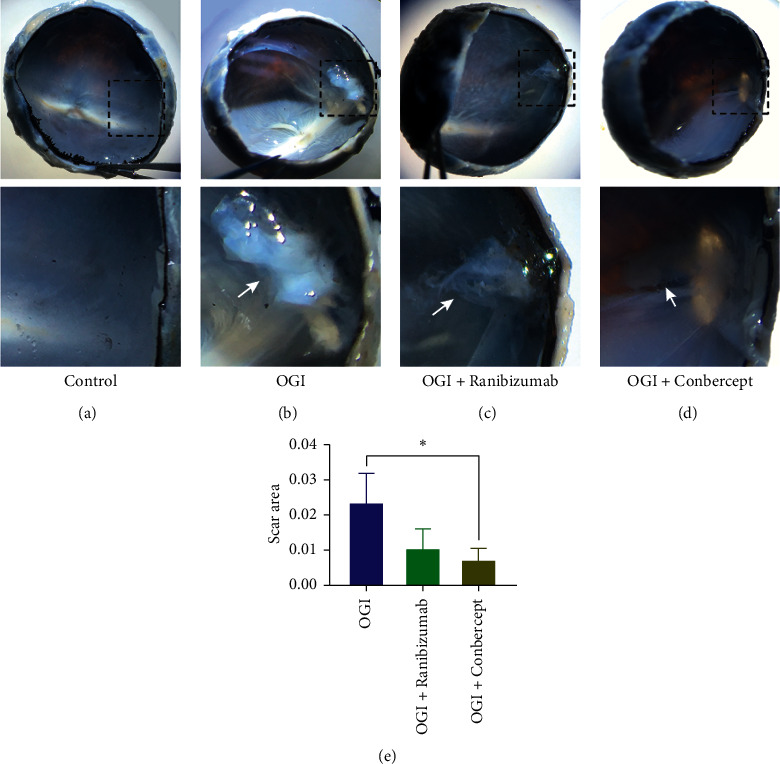
Gross view of rabbit eyes after open-globe injury. Ranibizumab or Conbercept was injected intravitreally at 0.5 hours after OGI; then eyeballs were enucleated for gross examination at 28 days after OGI. (a) Normal vitreous and retina. (b) Epiretinal porcelain scar (white arrow) formed at the wound site of rabbit eyes with OGI. Application of Ranibizumab (c) or Conbercept (d) inhibited the formation of epiretinal scar (white arrow) at the wound site. The bottom panel shows higher magnification of the boxed area of the original image above. (e) Quantification of the relative wound scar area as shown in (b)–(d). Data are mean ± SD, *n* = 3 animals per group. ^*∗*^*P* < 0.05 versus OGI by one-way ANOVA with Tukey's multiple comparisons test.

The maximal length of epiretinal scar at the wound site in rabbit eyes at 28 days after OGI was measured. Compared to eyes in control group, a large porcelain radial scar with tractional strands was observed at the wound site in rabbit eyes 28 days after OGI (Figure 3(b)). Ranibizumab showed an inhibitory effect in reducing the area of epiretinal scar, although statistical significance was not achieved (*P* = 0.1041) (Figures 3(c) and 3(e) and Table 2). In contrast, application of Conbercept dramatically decreased the length of epiretinal scar and inhibited the formation of vitreous strands induced by OGI (*P* = 0.048) (Figures 3(d) and 3(e) and Table 2).

### 3.4. Retinal Structure and Fibrosis Assessment

**Figure 4:**
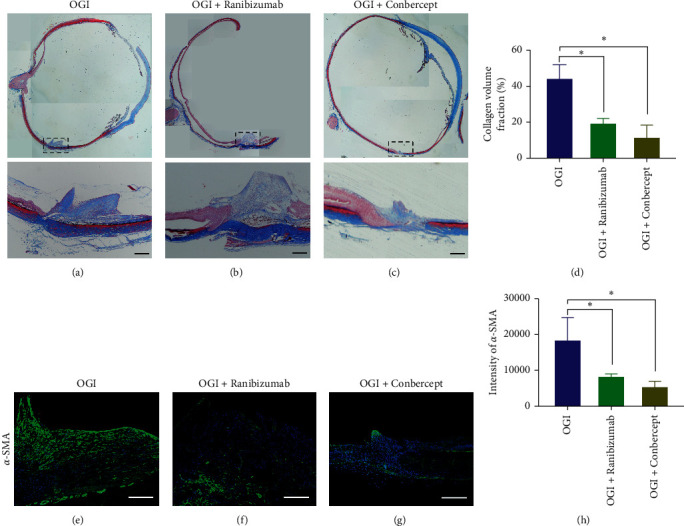
Assessment of retinal structure and fibrosis by Masson's staining and immunohistochemistry staining. ((a)–(c)) Masson's staining of rabbit eyes 28 days after OGI revealed bright blue staining at the wound site, indicating formation of fibrotic tissue. The bottom panel showed higher magnification of the boxed areas of the original image above. (d) Quantification of Masson's staining. (e–g) Representative images of *α*-SMA staining of retinal cryosections. Eyes with OGI showed strong fluorescent signals at the wound site, mitigated by intravitreal anti-VEGF treatments. (h) Quantification of *α*-SMA fluorescent intensities shown in (e–g). Data are mean ± SD, *n* = 3 animals per group. ^*∗*^*P* < 0.05 versus OGI by one-way ANOVA with Tukey's multiple comparisons test. Scale bar, 100 *μ*m ((a–c) and (e–g)).

Positive Masson's staining was observed at the wound site in OGI eyes at 28 days after injury (Figure 4(a)), which was mitigated by intravitreal injection of anti-VEGF agents (Figures 4(b)–4(d)). Similar results were further confirmed by staining of an *α*-SMA (Figures 4(e)–4(h)). These data show that both Ranibizumab and Conbercept reduced the size of fibrosis tissue at the wound site of OGI eyes, with Conbercept treatment showing a stronger effect.

### 3.5. Application of Anti-VEGF Agents Attenuated the Upregulation of VEGF and Other Cytokines in the Vitreous Induced by Open-Globe Injury

**Figure 5:**
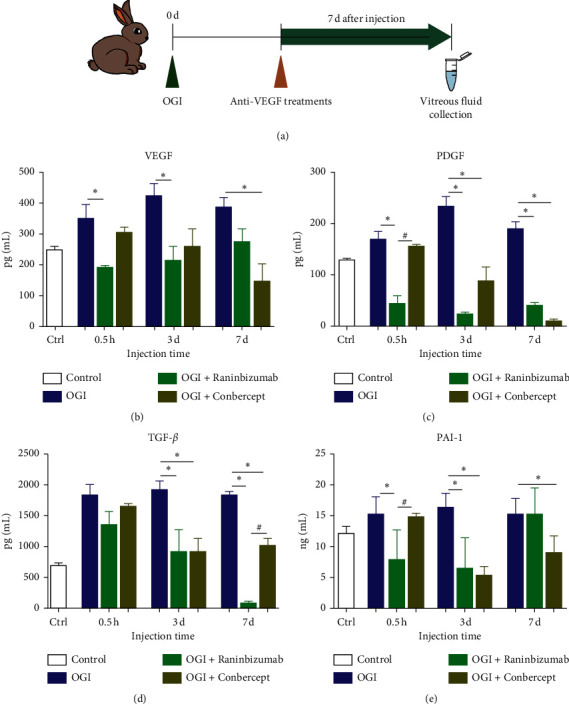
Application of anti-VEGF agents significantly decreased vitreous levels of VEGF and other cytokines induced by OGI. (a) A single dose of anti-VEGF agents was given at 0.5 h, 3 d, or 7 d after OGI, vitreous samples were collected 7 days thereafter, and vitreous cytokines were analyzed by ELISA. (b–e) Intravitreal injection of Ranibizumab and Conbercept significantly reduced the vitreous levels of VEGF and other cytokines induced by OGI. Data are mean ± SD, *n* = 5. ^*∗*^*P* < 0.05 versus control; ^#^*P* < 0.05, comparison between Ranibizumab and Conbercept groups by one-way ANOVA with Tukey's multiple comparisons test.

Compared to OGI group, treatment groups with anti-VEGF agents significantly reduced the vitreous levels of VEGF and other factors (Table S2). Specifically, Ranibizumab significantly reduced VEGF level when injected at 0.5 hours and 3 days (*P*_0.5*h*_ = 0.0164, *P*_3*d*_ = 0.0276) after OGI (Figure 5(b)), and inhibitory effect of Ranibizumab on vitreous levels of PDGF reached significance at all treatment time points (*P*_0.5*h*_ = 0.0002, *P*_3*d*_ < 0.0001, and *P*_7*d*_ < 0.0001) (Figure 5(c)). Vitreous TGF-*β* level was significantly decreased when Ranibizumab was injected at 3 and 7 days after OGI (*P*_3*d*_ = 0.0268, *P*_7*d*_ < 0.0001) (Figure 5(d)), and PAI-1 level was significantly decreased when Ranibizumab was injected at 0.5 hours and 3 days after OGI (*P*_0.5*h*_ = 0.0089, *P*_3*d*_ = 0.0025) (Figure 5(e)). In contrast to Ranibizumab, Conbercept significantly decreased VEGF level only when injected at 7 days after OGI (*P* = 0.0107) (Figure 5(b)) and decreased the levels of PDGF, TGF-*β*, and PAI-1 in vitreous significantly when injected at 3 days (*P*_*PDGF*_ = 0.0015, *P*_*TGF-β*_ = 0.0266, and *P*_*PAI-1*_ = 0.0011) and 7 days after OGI (*P*_*PDGF*_ < 0.0001, *P*_*TGF-β*_ < 0.0001, and *P*_*PAI-1*_ = 0.0298) (Figures 5(c)–5(e)).

We next compared the effectiveness between the two anti-VEGF agents. Ranibizumab and Conbercept showed similar effect in reducing vitreous VEGF levels in OGI eyes when injected at 0.5 hours and 3 days after OGI, but the lowest VEGF levels were achieved by injection of Conbercept at 7 days after OGI (VEGF_RBZ_ = 279.52 ± 77.61pg/ml, VEGF_CNB_ = 150.90 ± 106.06pg/ml, *P* = 0.1857) (Figure 5(b)). For PDGF, Conbercept showed a greater effect than Ranibizumab on decreasing PDGF level when injected at 7 days after OGI (PDGF_RBZ_ = 40.86 ± 10.66pg/ml, PDGF_CNB_ = 10.20 ± 6.70pg/ml, *P* = 0.3714) (Figure 5(c)). A lower vitreous level of TGF-*β* was found at 7 days in Ranibizumab injection group (TGF*ββ*_RBZ_ = 108.02 ± 16.51pg/ml, TGF*ββ*_CNB_ = 1020.25 ± 233.11pg/ml, *P* < 0.0001), while no significance was found between Conbercept and Ranibizumab at 0.5 hours or 3 days treatment time points (Figure 5(d)). Both Ranibizumab and Conbercept showed better effect on reducing PAI-1 levels at 3 days treatment time point (PAI-1_RBZ_ = 6.57 ± 4.92 ng/ml, PAI-1_CNB_ = 5.34 ± 1.41 ng/ml, *P* = 0.8487) (Figure 5(e)).

In summary, both Ranibizumab and Conbercept strongly inhibited the upregulation of VEGF and other cytokines in the vitreous induced by open-globe injury, and greater inhibitory effect of anti-VEGF agents can be achieved by injection of the agents at 3 or 7 days after OGI.

## 4. Discussion

The diverse nature of mechanical ocular trauma in humans prevents clinical assessment of the disease in controlled studies. In this study, we employed an OGI rabbit model to mimic the posterior penetrating injury. Although not fully representing human disease, this model recapitulates many features in PVR, including epiretinal scar and intravitreal proliferation process. This study attempts to investigate the role of anti-VEGF agents including Ranibizumab and Conbercept in OGI. We found that intravitreal application of anti-VEGF agents dramatically reduced the overexpression of growth factors and cytokines such as VEGF, PDGF, TGF-*β*, and PAI-1, and prevented the formation of epiretinal fibrosis at the wound site.

Aberrant concentrations of growth factors and cytokines in the vitreous have been implicated in the pathogenesis of PVR. Specifically, the role of TGF-*β* in inducing RPE EMT was considered the critical mechanism underlying PVR pathogenesis [23–26]. In addition, PDGFs have consistently been found to be elevated in PVR of animal models as well as human subjects [27, 28]. PAI-1 can bind and block tissue plasminogen activator (t-PA) and urokinase plasminogen activator (u-PA) and is implicated in tissue fibrosis in several diseases [29, 30]. In accordance with those reports, our study showed a significant upregulation of VEGF, PDGF, TGF-*β*, and PAI-1 for over 4 weeks after OGI; among these, TGF-*β* was the most overexpressed cytokine, which increased from 712.86 ± 75.19 pg/ml to 2105.09 ± 285.75 pg/ml after OGI. The elevation of these cytokines may contribute to the formation of epiretinal scar and fibrosis at the wound site. In our study, application of anti-VEGF agents significantly attenuated the upregulation of VEGF, PDGF, TGF-*β*, and PAI-1 induced by OGI, and greater effect can be achieved when the agents were applied at day 3 or day 7 after OGI. Moreover, application of anti-VEGF agents at 0.5 hours after OGI could effectively prevent the epiretinal scar formation at the wound site. Overall, these results suggest a potential beneficial effect of anti-VEGF agents on improving PVR progression by decreasing intraocular proliferative and profibrotic factors.

Ocular trauma induces a retinal wound-healing process containing three phases: 4 to 6 days of inflammatory phase, followed by fibroplasia or collagen production phase for 2 to 4 weeks and tissue remodeling phase that lasts for years [31]. In our study, inflammatory cytokines and coagulation factors like VEGF, PDGF, TGF-*β*, and PAI-1 were all significantly elevated within 3 to 10 days after OGI, indicating inflammatory reactions and immediate platelet activation after OGI. At the end of inflammatory phase, fibroblasts proliferate and migrate into the wound site and start to produce collagen for wound repair, which is characterized as fibroplasia phase [32]. In our study, VEGF, PDGF, and PAI-1 increased significantly at day 10 but reduced at day 14; then they increased again at days 21–28. This may reflect the two stages of response of these growth factors, suggesting a contribution of these factors in promoting fibroblasts proliferation and collagen production. In accordance with our study, Wong et al. reported persistent elevation of VEGF and PDGF for 4 weeks in an experimental PVR model, and their vitreous levels were significantly associated with PVR severity [33]. Altogether, these data suggest that growth factors and cytokines are important contributors to PVR pathogenesis and decreasing these factors may inhibit the proliferation and fibrotic process associated with PVR development.

Ranibizumab and Conbercept are both effective anti-VEGF agents commonly used in treating retinal diseases such as CNV, diabetic macular edema (DME), retinal vein occlusion (RVO), and pathologic myopia (PM) [34–36]. Ranibizumab is a recombinant humanized monoclonal immunoglobulin G1 (IgG1) *κ*-isotype Fab fragment that can neutralize all isoforms of VEGF-A. Conbercept is a fusion protein composed of the ligand binding elements of extracellular domains of VEGF receptors 1 and 2 fused to the Fc portion of human IgG1. It could bind different VEGF-A isoforms and placental growth factor (PLGF). In our study, intravitreal application of Ranibizumab and Conbercept effectively reduced the expression of VEGF after OGI. Moreover, anti-VEGF treatments also attenuated the upregulation of PDGF, TGF-*β*, and PAI-1 induced by OGI, suggesting a wider spectrum of action for anti-VEGF agents. The exact mechanism of how anti-VEGF agents downregulates these cytokines is not clear and may be a direct effect of VEGF-A neutralization. Previous studies have shown that VEGF-A competitively inhibits PDGF-dependent binding and activation of PDGFR [37, 38]; therefore, downregulation of VEGF may promote PDGF-PDGFRs binding and its subsequent internalization and degradation, resulting in decreased PDGF levels in the vitreous. Another study demonstrated that application of Ranibizumab protected rabbits from developing PVR via suppressing PDGFR/PI3K/Akt signaling [18]. Furthermore, previous studies demonstrated that VEGF can regulate the mRNA expression of PAI-1 in microvascular endothelial cells [39]; thus, neutralization of VEGF may decrease PAI-1 expression. Finally, the reduced vascular permeability and leakage, and possibly reduced immune cell infiltration and inflammation in OGI eyes after intravitreal anti-VEGF treatments, may account for the inhibitory action of anti-VEGF agents on other cytokines. In brief, our data suggest that intervention of anti-VEGF agents after ocular trauma may help restore retinal homeostasis by reducing the inflammatory and fibrotic response associated with the injury.

In accord with our study, several other studies have reported the effect of intravitreal anti-VEGF agents including Ranibizumab in reducing the extent of fibrosis and the development of PVR in experimental PVR models [18, 19, 38]; however, the role of Conbercept in PVR has not been analyzed. Here, we report in our study that both Ranibizumab and Conbercept can effectively decrease the vitreous levels of growth factors and cytokines in rabbit eyes with OGI, and intravitreal Conbercept showed stronger effect in preventing epiretinal scar formation at the wound site. Conbercept is constructed as a broad-spectrum anti-VEGF agent, similar to VEGF-trap, which binds all isotypes of VEGF-A and PLGF. Moreover, the half-life (*t*_1/2_) of Conbercept in rabbit vitreous is longer than Ranibizumab (4.2 days versus 2.9 days) [40, 41], therefore, Conbercept may provide sustained effects in reducing retinal inflammation and protecting vascular permeability and hence may be more beneficial in reducing the risk of PVR development.

In the current study, we employed an OGI rabbit model that successfully developed intravitreal proliferation and epiretinal scar at the wound site; however, a severe manifestation of PVR such as tractional retinal detachment was not observed. PVR is a complex, multifactorial disease that involves multiple risk factors; for example, advanced PVR is usually accompanied with severe RD, giant retinal breaks, and intraocular hemorrhage [3, 42]. By applying retinal injuries combined with intravitreal injection of fibroblasts and platelet-rich plasma, previous studies have successfully induced PVR complicated with vitreous membrane and tractional RD in rabbits [18, 38], whereas, in our study, without additional intraocular proliferative factors, only ocular injury may not be sufficient to induce the severe manifestations of the disease. Therefore, the OGI model we employed might be useful to study the pathogenesis of early PVR. A recent meta-analysis analyzed the effect of bevacizumab in PVR patients with RD and reported no significant effect of bevacizumab on inhibiting PVR progression [43]. Several confounding factors might affect the efficacy of anti-VEGF agents, where, among them, the timing of anti-VEGF administration was considered a crucial factor. As discussed in the paper, application of anti-VEGF agents may be insufficient to stop the disease progress once the cascade of inflammation processes exceeds a threshold. Therefore, the efficacy of anti-VEGF treatments should be evaluated in earlier PVR or patients at high risk of PVR. Our study demonstrated that, in an animal model representing early PVR, application of anti-VEGF agents effectively inhibited the intravitreal proliferation and formation of fibrotic scar at the wound site induced by OGI, indicating a beneficial effect of early anti-VEGF treatment in preventing PVR development.

## 5. Conclusions

Intravitreal application of anti-VEGF agents significantly reduced the growth factors and cytokines including VEGF, PDGF, TGF-*β*, and PAI-1 in vitreous fluid of rabbit eyes after OGI. Both Ranibizumab and Conbercept had an obvious effect on inhibiting the formation of epiretinal scar at the wound site, while Conbercept showed stronger effect on preventing the fibrotic scar formation, probably due to its broad-spectrum anti-VEGF characteristic and longer half-life. These results suggest that anti-VEGF treatments may work as potential prophylaxis to prevent the progression of posttraumatic PVR.

## Figures and Tables

**Table 1:** Intravitreal proliferation stage classified via proliferative responses and corresponding B scan manifestations.

Intravitreal proliferation stage	Proliferative responses^†^	B scan manifestations^†^
Stage 0	No proliferative response	Normal
Stage 1	Vitreous haze, vitreous strands	Vitreous opacity, without membranous echo connected to the retina
Stage 2	Epiretinal membrane formation with retinal folds	Membranous echo connected to the retina, without local retinal detachment
Stage 3	White dense membrane covering the retina with localized retinal detachments and retinal folds	Surface of the retina not smooth; significant vitreous traction and retinal detachment

^†^Proliferative responses and corresponding B scan manifestations were summarized according to previous studies.

**Table 2:** Changes of maximal length of epiretinal scar after anti-VEGF treatments at 28 days after OGI.

Group	Epiretinal scar length	*P* value∗
	( $\bar{\chi }\pm \text{SD}$, *μ*m)	
Control	0	—
OGI	503 ± 82.44	—
OGI + Ranibizumab	355 ± 43.66	0.244
OGI + Conbercept	250.33 ± 36.02	0.048

∗*P* values were compared with OGI group by one-way ANOVA with Tukey's multiple comparisons test. Data are mean ± SD, *n* = 3.

## Data Availability

The relevant data of this study are available from the corresponding author upon request.

## Supplementary Materials

Supplementary Materials Table S1: changes of vitreous factors in rabbit after OGI from baseline to 28 d after injury. Table S2: levels of vitreous factors in rabbit models of OGI after application of anti-VEGF agents.
