# Supplementary material for: The Role of Intravitreal Anti-VEGF Agents in Rabbit Eye Model of Open-Globe Injury
Source: J Ophthalmol. 2021 Apr 15;2021:5565178. doi: 10.1155/2021/5565178 (PMC8064804; doi:10.1155/2021/5565178)
Supplement: Supplementary Materials — Table S1: changes of vitreous factors in rabbit after OGI from baseline to 28 d after injury. Table S2: levels of vitreous factors in rabbit models of OGI after application of anti-VEGF agents. [file 5565178.f1.docx]

**Table S1. Changes of vitreous factors in rabbit after OGI from baseline to 28d post injury.**

| **Intraocular factors** | **Control Group** | **0.5d** | | **1d** | | **3d** | | **7d** | | **10d** | | **14d** | | **21d** | | **28d** | |
| --- | --- | --- | --- | --- | --- | --- | --- | --- | --- | --- | --- | --- | --- | --- | --- | --- | --- |
|  |  | **OGI Group** | ***P* value** | **OGI Group** | ***P* value** | **OGI Group** | ***P* value** | **OGI Group** | ***P* value** | **OGI Group** | ***P* value** | **OGI Group** | ***P* value** | **OGI Group** | ***P* value** | **OGI Group** | ***P* value** |
| VEGF (pg/ml) | 252.24±  27.49 | 294.83±  101.08 | 0.994 | 238.35±  71.33 | 1.000 | 374.83±  89.67 | 0.270 | 351.83±  106.40 | 0.540 | 425.50±  90.95 | 0.028* | 362.80±  85.50 | 0.400 | 448.53±  99.87 | 0.008* | 489.83±  77.32 | 0.0018 |
| PDGF (pg/ml) | 136.82±  1.71 | 163.46±  39.25 | 0.997 | 144.54±  24.79 | 1.000 | 183.48±  48.86 | 0.903 | 171.16±  42.72 | 0.983 | 235.62±  49.99 | 0.129 | 189.42±  40.30 | 0.829 | 258.80±  34.53 | 0.028* | 294.51±  47.27 | 0.003* |
| TGF-β (pg/ml) | 712.86±  75.19 | 1685.97±  219.94 | 0.000* | 1823,77±  196.46 | 0.000* | 1837.03±  200.85 | 0.000* | 1834.04±  381.88 | 0.000* | 1924.91±  285.02 | 0.000* | 1845.74±  113.29 | 0.000* | 1939.60±  223.47 | 0.000* | 2105.09±  285.75 | 0.000* |
| PAI-1 (ng/ml) | 12.15±  1.06 | 13.37±  1.79 | 0.981 | 13.19±  1.58 | 0.993 | 15.72±  1.65 | 0.097 | 15.40±  2.67 | 0.170 | 16.30±  2.25 | 0.030* | 15.40±  2.53 | 0.169 | 17.16±  1.77 | 0.004* | 16.96±  2.68 | 0.007* |

* Statistically significant compared to control group.

Data were shown as mean ± SD, n$\geq$5 for each time point.

**Table S2. Levels of vitreous factors in rabbit models of OGI after application of anti-VEGF agents.**

| **Intraocular factors** | **Control** | **anti-VEGF agents injected 0.5h post OGI** | | | | |  | **anti-VEGF agents injected 3d post OGI** | | | | |  | **anti-VEGF agents injected 7d post OGI** | | | | |
| --- | --- | --- | --- | --- | --- | --- | --- | --- | --- | --- | --- | --- | --- | --- | --- | --- | --- | --- |
|  |  | **OGI** | **OGI+ Ranibizumab** | ***P* value^a^** | **OGI +**  **Conbercept** | ***P* value^b^** |  | **OGI** | **OGI +**  **Ranibizumab** | ***P* value^c^** | **OGI +**  **Conbercept** | ***P* value^d^** |  | **OGI** | **OGI +**  **Ranibizumab** | ***P* value^e^** | **OGI +**  **Conbercept** | ***P***  **value^f^** |
| VEGF (pg/ml) | 252.24±  27.49 | 351.83±  106.40 | 194.45±  13.44 | 0.016 | 306.03±  44.95 | 0.572 |  | 425.50±  90.95 | 216.73±  95.01 | 0.028 | 262.63±  116.09 | 0.084 |  | 390.78±  67.28 | 279.52±  77.61 | 0.266 | 150.90±  106.06 | 0.011 |
| PDGF (pg/ml) | 135.25±  2.20 | 171.16±  38.21 | 44.57±  28.00 | 0.000 | 155.58±  4.60 | 0.650 |  | 235.62±  44.71 | 22.20±  3.27 | 0.000 | 88.47±  47.42 | 0.002 |  | 189.42±  36.05 | 40.86±  10.66 | 0.000 | 10.20±  6.70 | 0.000 |
| TGF-β (pg/ml) | 712.86±  75.19 | 1834.04±  381.88 | 1363.58±  352.82 | 0.099 | 1661.43±  83.71 | 0.583 |  | 1924.91±  285.02 | 926.68±  684.07 | 0.027 | 925.28±  426.39 | 0.027 |  | 1845.74±  113.29 | 108.02±  16.51 | 0.000 | 1020.27±  233.11 | 0.000 |
| PAI-1 (ng/ml) | 12.15±  1.06 | 15.40±  2.67 | 7.90±  4.85 | 0.009 | 14.93±  0.52 | 0.968 |  | 16.30±  2.25 | 6.57±  4.92 | 0.003 | 5.34±  1.41 | 0.001 |  | 15.40±  2.53 | 15.32±  4.12 | 0.999 | 9.14±  2.63 | 0.030 |

^a^ and ^b^ compared to the corresponding OGI group in anti-VEGF agents injected 0.5h post OGI group, ^c^ and ^d^ compared to the corresponding OGI group in anti-VEGF agents injected 3d post OGI group, ^e^ and ^f^ compared to the corresponding OGI group in anti-VEGF agents injected 7d post OGI group.

Vitreous fluid samples were collected 7 days after injection of anti-VEGF agents. Data were shown as mean ± SD, n=5.
